# Thyroid metastasis from ovarian clear cell carcinoma

**DOI:** 10.1530/EDM-24-0086

**Published:** 2024-12-19

**Authors:** Rika Sasaki, Haruhiko Yamazaki, Eita Kumagai, Soji Toda, Aya Saito

**Affiliations:** ^1^ Department of Breast and Thyroid Surgery, Yokohama City University Medical Center, Yokohama, Kanagawa, Japan; ^2^ Department of Pathology, Yokohama City University Medical Center, Yokohama, Kanagawa, Japan; ^3^ Department of Surgery, Yokohama City University School of Medicine, Yokohama, Kanagawa, Japan

**Keywords:** Thyroid, Metastasis, Ovarian, Carcinomas

## Abstract

**Summary:**

A 56-year-old woman with cervical pain with a history of ovarian clear cell carcinoma stage IIIC was admitted to a primary care doctor. Ultrasonography revealed a microhyperechoic nodule in the thyroid gland and cervical lymph node enlargement, and fine-needle aspiration was performed. The results showed malignancy, and she was admitted to our hospital. The differential diagnoses included primary thyroid neoplasms and thyroid metastases from ovarian clear cell carcinoma. A needle biopsy of the thyroid gland was performed. Immunohistochemistry revealed that the tumor cells were positive for cytokeratin AE1/AE3, hepatocyte nuclear factor-1-beta and PAX8 and negative for thyroglobulin and thyroid transcription factor-1. Therefore, we diagnosed the patient with thyroid metastasis from ovarian clear cell carcinoma. There were no compressive symptoms at the time of the visit to our hospital, and surgery was considered unnecessary. Systemic treatment for ovarian clear cell carcinoma was continued. Three months later, she died of a stroke due to Trousseau’s syndrome.

**Learning points:**

- Metastasis of ovarian carcinoma to the thyroid gland is extremely rare.
- Using histology and immunostaining, we were able to accurately diagnose thyroid metastasis of ovarian clear cell carcinoma.

## Background

Metastasis to the thyroid gland is uncommon, with a reported frequency between 1.4 and 2.5% among all thyroid malignancies (1). Kidney and lung carcinomas are the most common primary malignant neoplasms that metastasize to the thyroid (1). In contrast, metastases from primary ovarian cancer are extremely rare, with a prevalence of 3% (2). We herein report a case of ovarian clear cell carcinoma that metastasized to the thyroid gland.

## Case presentation

**Figure 1:**
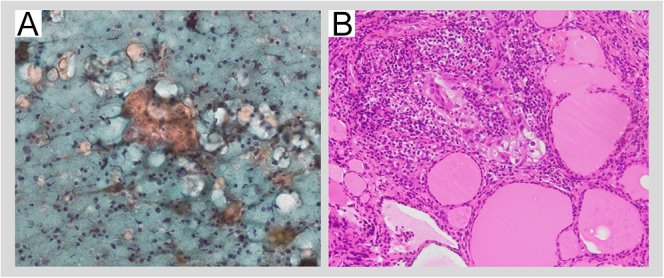
Pathological findings. (A) Fine-needle aspiration cytology showing malignant cells with enlarged nuclei, prominent nucleoli and clear cytoplasm, presenting an atypical appearance compared to usual papillary carcinoma. (b) Core needle biopsy revealing neoplastic cells with enlarged nuclei and prominent nucleoli.

A 56-year-old woman with cervical pain was admitted to a primary care doctor. The patient underwent surgery for ovarian clear cell carcinoma stage IIIC in the gynecology department of our hospital 2 years previously. Chemotherapy was subsequently administered for the recurrent lesions. Ultrasonography performed by a previous doctor revealed a microhyperechoic nodule in the thyroid gland and cervical lymph node enlargement. Fine-needle aspiration (FNA) of the thyroid nodule and cervical lymphadenopathies were performed under ultrasonography guidance, which revealed the presence of cells with enlarged nuclei and prominent nucleoli and cells with clear cytoplasm (Fig. 1A). This finding was considered malignant, and she was admitted to our hospital with suspected thyroid cancer. A physical examination revealed a small, non-tender thyroid gland upon palpation. The blood test results were as follows – white blood cell: 6.02 × 10^3^ μL; hemoglobin: 8.2 g/dL; mean corpuscular volume: 96.4 fL; platelet: 327 × 10^3^/μL; albumin: 2.4 g/dL; aspartate aminotransferase: 17 U/L; alanine aminotransferase: 13 U/L; creatinine: 0.46 mg/dL; blood urea nitrogen (BUN): 13 mg/dL; Na: 140 mmol/L; K: 3.9 mmol/L; C-reactive protein: (CRP): 14.082 mg/dL; carcinoembryonic antigen (CEA): <1.8 ng/mL; cancer antigen 125 (CA125): 48 U/mL; CA19-9: 27 U/mL; thyroid stimulating hormone (TSH): 0.013 μIU/mL; Tg: 32.2 ng/dL; FT3: 3.03 pg/mL; TgAb: 10.2 ng/mL; and FT4: 1.43 ng/mL.

**Figure 2:**
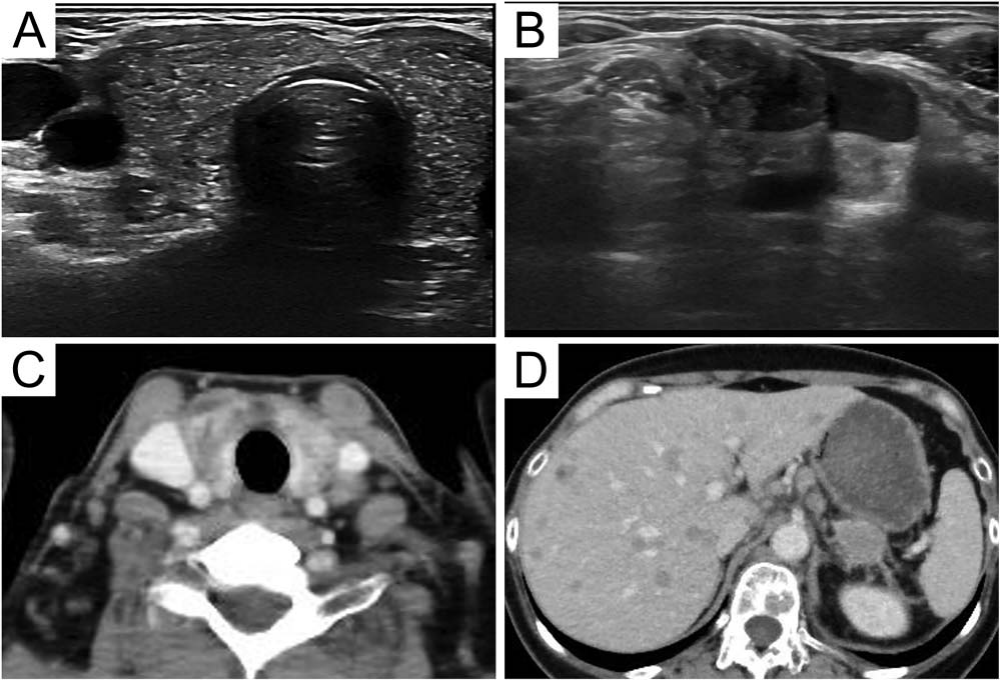
Imaging findings. (A) Ultrasonographic image showing scattered dot-like high echoes in the thyroid gland. (B) Ultrasonographic image showing enlarged cervical lymph nodes. (C) CT image showing a low-density area in the thyroid gland, without signs of airway invasion. (D) CT image demonstrating liver and bone metastases.

Ultrasonography showed scattered dot-like high echoes in the thyroid gland and enlarged lymph nodes in the neck (Fig. 2A and B). The Thyroid Imaging Reporting and Data System grade was 6 points (composition was solid: 2 points, echogenicity was hyperechoic: 1 point, shape could not be assessed, margin was smooth: 0 point, and echogenic foci were punctate echogenic foci: 3 points). CT showed a low-density area in the thyroid gland. However, no airway invasion was found (Fig. 2C).

## Investigation

**Figure 3:**
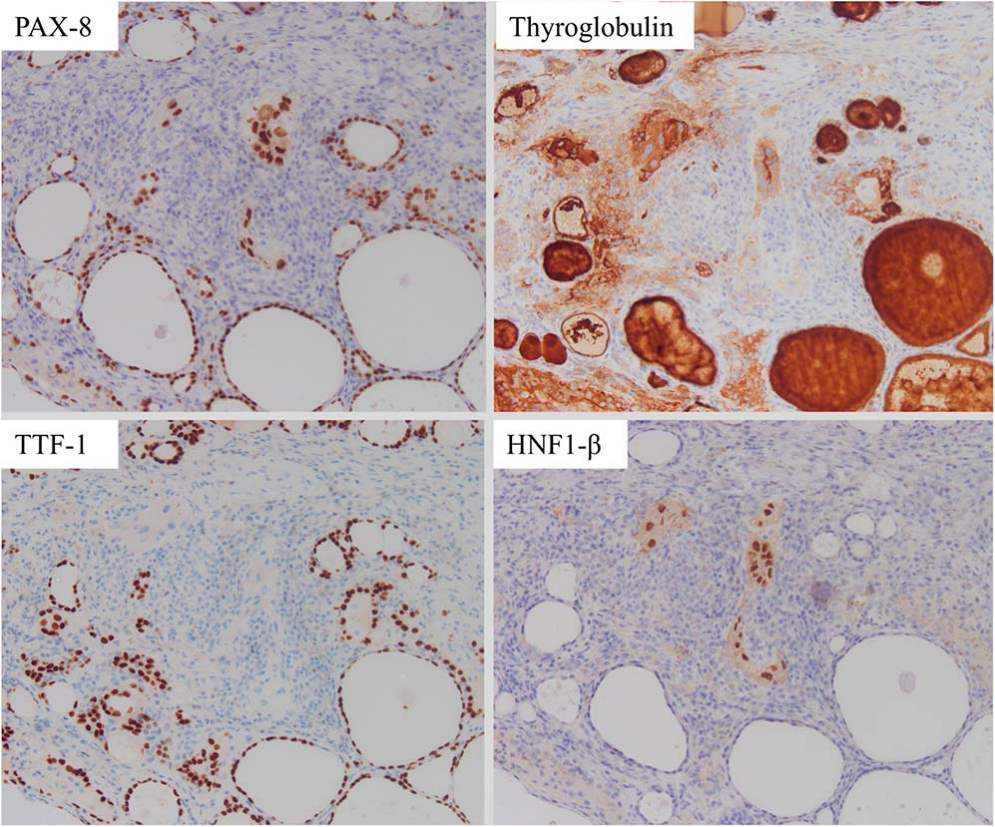
Immunohistochemical examination showing the thyroid tumor to be positive for PAX8 and hepatocyte nuclear factor-1-beta (HNF1β) but negative for thyroglobulin and TTF-1. HNF1β has been reported to be useful in ovarian clear cell carcinoma.

The differential diagnoses included primary thyroid neoplasms and thyroid metastases from ovarian clear cell carcinoma. Core needle biopsy (CNB) of the thyroid gland was performed. A pathological examination revealed the presence of neoplastic cells with enlarged nuclei and prominent nucleoli (Fig. 1B). Immunohistochemistry revealed that the tumor cells were positive for cytokeratin AE1/AE3, hepatocyte nuclear factor-1-beta (HNF1β) and PAX8 and negative for thyroglobulin and thyroid transcription factor-1 (TTF-1) (Fig. 3). Therefore, we diagnosed the patient with thyroid metastasis from ovarian clear cell carcinoma.

## Treatment

Following consultation with gynecologists, systemic treatment for ovarian clear cell carcinoma was continued. There were no compressive symptoms at the time of the visit to our hospital, and surgery was considered unnecessary.

## Outcome and follow-up

The patient subsequently developed liver and bone metastases (Fig. 2D). Three months after the diagnosis of metastatic thyroid cancer, the patient died of a stroke due to Trousseau’s syndrome.

## Discussion

The thyroid gland is a rare site of metastasis in comparison with other organs. Several hypotheses have been proposed: i) thyroid glands have a rich blood supply, which may prevent cancer cell colonization; ii) the high oxygen environment in thyroid tissue may inhibit the proliferation of cancer cells; and iii) tumor cells may be trapped in the lungs during the process of hematogenous metastases (3).

The most common primary sites were the kidney (23.7%), the lung (21.4%), craniocervical region (12.6%), breast (10.7%), esophagus (7.0%) and colon (6.8%) (4). Thyroid metastases from the ovaries and uterus are extremely rare, with a frequency of 3%. Synchronous metastases occurred in 34% of cases. In contrast, asynchronous metastases have been reported in 60% of cases (5). In the present case, the patient was considered to have developed asynchronous metastasis because it was found during treatment for recurrent ovarian cancer.

Ultrasonographic images of thyroid metastases can vary from solid, ill-defined margins to dot-like high echoes in the thyroid gland (6). Scattered dot-like high echoes were observed in this case. Therefore, it is necessary to differentiate it from the diffuse sclerosing variant of papillary thyroid carcinoma (7). The diagnostic accuracy of a cytological diagnosis is approximately 50% (8). A CNB and immunohistochemistry are useful for differentiation. In patients with a history of malignancy, the possibility of metastatic thyroid tumors should be considered.

Metastasis of ovarian carcinoma to the thyroid gland is very rare, with only two cases described in the relevant literature (9, 10). Vaslamatzis reported a case of solitary thyroid metastasis from a stage IV high-grade serous ovarian carcinoma. The patient was treated with surgery and combined chemotherapy, which resulted in a complete remission. Four years after the surgery, biochemical relapse with increasing CA125 values was observed, so PET–CT was performed. Pathological uptake was observed in the thyroid lesion in the right lobe. A fine-needle biopsy of the thyroid lesion showed metastatic infiltration from the same ovarian carcinoma. The patient underwent a right thyroid lobectomy and received combined chemotherapy again. The patient remains in complete remission (9). Ji and coworkers reported a case of thyroid metastasis from stage IC ovarian clear cell carcinoma. One and a half years after surgery, a mass localized in the right lobe of the thyroid appeared. FNA of the thyroid tumor was performed, and a metastatic thyroid tumor was suspected. Immunocytochemistry was performed. The tumor cells were positive for CA125 and HNF1β but negative for thyroglobulin, TTF-1 and PAX8. The patient was diagnosed with ovarian clear cell carcinoma with metastasis to the thyroid gland (10). In our case, thyroid metastasis was found 2 years after the ovarian surgery. Performing FNA and proceeding to immunocytochemistry, we diagnosed the patient with thyroid metastasis from ovarian clear cell adenocarcinoma (Table 1).

**Table 1:** Characteristics of cases with TM from OC.

Case no.	Study	Year of publication	Age at diagnosis, years		Original diagnosis	Methods of diagnosing TM	ICC/IHC	Treatment for TM	Outcome
			OC	TM					
1	Vaslamatzis *et al.* (9)	2018	51	55	Serous ovarian carcinoma	FNB	None	Right thyroidectomy, chemotherapy	Complete remission
2	Ji *et al.* (10)	2021	42	44	Clear cell carcinoma	FNA	ICC: CA125 (+), HNF1β (+), Tg (−), TTF-1 (−), PAX8 (−)	Not stated	Not stated
3	Our case	–	54	56	Clear cell carcinoma	CNB	IHC: CK AE/AE3(+), HNF1β (+), PAX8(+), Tg (−), TTF-1(−)	Chemotherapy	Died 3 months after the diagnosis of MTC

FNA, fine-needle aspiration; FNB, fine-needle biopsy; CA125, cancer antigen 125; CK AE/AE3, cytokeratin AE1/AE3; CNB, core needle biopsy; HNF1β, hepatocyte nuclear factor-1-beta; ICC, immunocytochemistry; IHC, immunohistochemistry; MTC, metastatic thyroid cancer; OC, ovarian cancer; PAX8, paired box 8; Tg, thyroglobulin; TM, thyroid metastasis; TTF-1, thyroid transcription factor-1.

As treatment strategies differ between primary thyroid neoplasms and metastatic thyroid cancer, it is important to differentiate between them. TTF-1 and PAX8 are thyroid-specific immunohistochemical markers. PAX8, CDX2 and SATB2 are useful for the diagnosis of mucinous ovarian carcinoma, while PAX8 and WT1 are useful for the diagnosis of serous ovarian carcinoma (https://www2.tri-kobe.org/nccn/guideline/occult/japanese/occult.pdf; accessed on 5 November 2024). HNF1β has also been reported to be useful in ovarian clear cell adenocarcinoma (11). In our case, the tumor cells were negative for thyroglobulin and TTF-1 but positive for PAX8 and HNF1β. Therefore, it was feasible to diagnose ovarian metastasis of thyroid cancer.

Surgery may be considered for metastatic thyroid tumors because of their rapid growth rate and the possibility of invasion into the surrounding organs (12). Surgery is indicated in patients whose primary tumor is under control and who have no metastasis to other organs (13). Most patients with metastasis to the thyroid had poor outcomes, with a reported survival time of 2 weeks to 15 months after the diagnosis(14). The goal of surgery is not to prolong the prognosis but to relieve compressive symptoms in patients with disseminated disease (15). Therefore, it is important to consider the timing of thyroidectomy. In our case, there were no compressive symptoms at the time of the visit to our hospital, and surgery was considered unnecessary. In this case, after the diagnosis of thyroid metastasis, multiple liver metastases rapidly appeared. Therefore, thyroid metastasis may have occurred as a result of progression of the primary disease.

## Conclusions

We herein described the case of a patient with thyroid metastasis from ovarian clear cell carcinoma. Using histology and immunostaining, we were able to accurately diagnose thyroid metastasis of ovarian clear cell carcinoma.

## Declaration of interest

The authors declare that there is no conflict of interest that could be perceived as prejudicing the impartiality of the work reported.

## Funding

This work did not receive any specific grant from any funding agency in the public, commercial or not-for-profit sector.

## Patient consent

Written informed consent for publication of her clinical details and clinical images was obtained from the patient. A copy of the written consent is available for review by the Editor-in-Chief of this journal.

## Author contribution statement

RS prepared the manuscript. HY and ST cared for the patient. EK performed a pathological diagnosis. AS comprehensively supervised this case report. All authors read and approved the final manuscript.
